# Epidermolysis bullosa pruriginosa: A case report of two first cousins

**DOI:** 10.12669/pjms.39.5.6764

**Published:** 2023

**Authors:** Maria Zahoor

**Affiliations:** 1 Dr. Maria Zahoor MSc Clinical Dermatology (University of Hertfordshire, UK) M.C.P.S (Paediatrics), M.B.B.S (Dow Medical College) Unit III, Department of Paediatric Medicine, National Institute of Child Health, Rafique S.J Shaheed Road, Karachi, 75510, Pakistan

**Keywords:** Genodermatoses, Epidermolysis bullosa pruriginosa, pruritus

## Abstract

Genodermatoses are quite frequent in developing countries where consanguinity is common but are usually under reported and undiagnosed. Main reason being lack of accessibility to tertiary health care facilities for people of rural areas as evident in case below. Genetic counselling and pre natal testing is of utmost importance in affected families. Epidermolysis bullosa pruriginosa (EBP) is a rare and less recognized variant of dystrophic epidermolysis bullosa. Reporting the case of two first cousins who presented with intensely pruritic skin lesions since infancy along with the history of siblings with skin problems. EBP provided a unifying diagnosis.

## INTRODUCTION

Epidermolysis Bullosa Pruriginosa (EBP) is a rare subtype of Dystrophic EpidermolysisBullosa.^1^ Hall mark of this condition is intense pruritis with prurigo like skin lesionsalong with lichenification and scarring.^1^ In literature review, the age of presentation is quite variable. This skin condition was properly documented first time in 1994 by McGrath in the form of a case study of eight patients with an unusual expression of dystrophic epidermolysis bullosa.

Five cases were sporadic, three gave a history of familial involvement with autosomal dominant inheritance in two cases and recessive in the other.^2^ Unlike other genodermatoses, majority of case reports of epidermolysis bullosa pruriginosa comprises of adult population. This case is being reported due to its rarity and familial clustering. There is paucity of literature on this different presentation of epidermolysis bullosa in paediatric population and also it is very rarely reported from Pakistan.

## CASE PRESENTATION

Two male children (first cousins), nine years and six years old respectively were referred to our hospital for evaluation of their skin lesions which were present since first year of their lives. As they belong to one of the remote rural area with lack of basic health facilities and specialist care, no proper diagnosis was made and some herbal creams were randomly being applied. According to parents, initially children had few blisters on peripheral limbs which decreased with time followed by itchy skin lesions.

Patients were products of consanguineous marriages. Parents did not have any skin condition and were not able to recall about skin problems in forefathers. However, two siblings (one brother and one sister) of younger patient and one brother of elder patient died in infancy and they had blistering skin lesions which remained undiagnosed. Rest of the siblings (one brother of younger patient and two sisters of elder patient) were alive and healthy with no skin problem. On examination, both children were severely anaemic. Their height and weight wasbelow 10^th^ centile for their ages.

**Fig.1:**
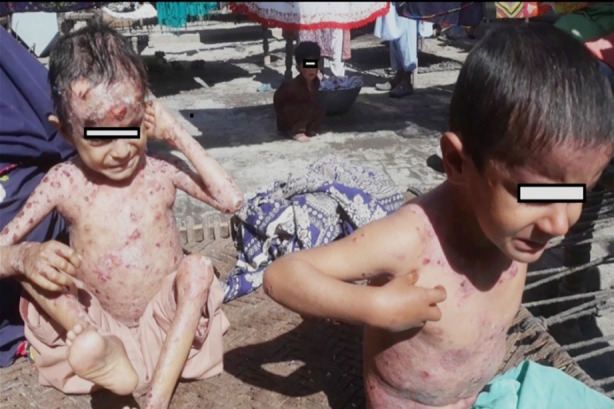


**Fig.2:**
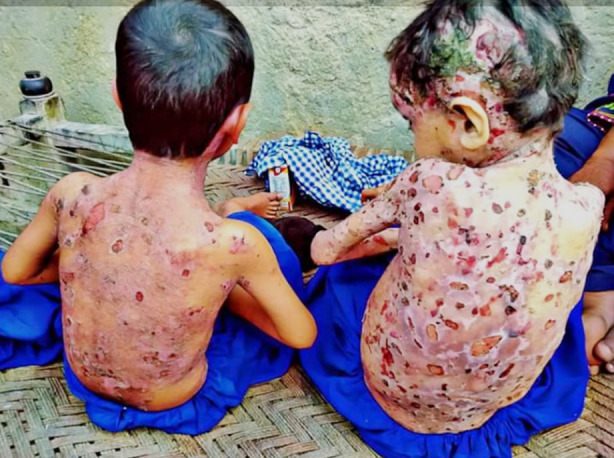


They were badly scratching their skin lesions whichlooked like nodular prurigo type plaques at the back, abdomen and limbs Fig.1. Patches of violaceous scarring were also there Fig.2. Pus was also oozing from some excoriated skin lesions. No intact bullae was observed in both patients. Face and scalp was also involved in one child with areas of hair loss at the site of lesions. Dystrophic nail changes were evident in more than 50% of the nails in both children. Mucous membranes were not involved.

Skin biopsy of both patients was quite similar. It showed skin tissue with separatedepidermis. The deeper dermis revealed infiltrate comprising of eosinophils, histiocytes,mast cells and lymphocytes as also highlighted on immunochemical stains CD3, CD117and CD68. Sections stained with IgG,IgA, IgM, C3 and C1q using direct immunofluorescence technique were negative in both patients. Serum IgE levels were within normal range.

Keeping in view strong clinical history and examination, diagnosis of epidermolysisbullosa pruriginosa was made. Genetic counselling of parents was done and importance of pre-natal testing in next pregnancies was explained. Packed red blood cells were transfused. Nutritional deficiencies were addressed.

Molecular mutational analysis is not performed as this facility is not available at our hospital and family was non affording to get it done privately from somewhere else. Topical emollients comprising of paraffin and glycerine were prescribed. Skin swabs for pus culture were sent and antibiotics were administered accordingly.When infection resolved, moderate potency topical steroid was prescribed for a short period. Children were discharged as they were stable. In follow up visits, steroids were gradually replaced by topical 0.03% tacrolimus which was also helpful in relieving intense pruritis.

## DISCUSSION

Epidermolysis bullosa pruriginosa is sometimes a difficult diagnosis due to variability in age of onset, rarity of intact bullae, histologic ambiguities and resemblance to acquired inflammatory dermatoses.^3^ This variant of dystrophic epidermolysis bullosa may not manifest clinically until adulthood and often misdiagnosed as other acquired skin conditions like nodular prurigo, lichen planus, lichen simplex etc.^4^ Mutations in COL7A1 gene encoding the anchoring fibril protein, type VII collagen is linked to Epidermolysis bullosa pruriginosa but genotype-phenotype correlation is not clear.^4^

Microscopic studies indicate the findings of dystrophic epidermolysis bullosa withunusually high density of collagen bundles in some cases and it has been postulated that itchiness might be due to an abnormal dermal reactivity in some patients to their inherited skin disorder.^1^ Molecular heterogeneity in epiderdermolysis bullosa pruriginosa with glycine substitution mutations, a deletion mutation and compound heterozygosity for a frameshift mutation along with splice site mutation are involved in giving rise to this variant of dystrophic epidermolysis bullosa.^5^

Delayed onset of clinical features in some patients yet remains unclear.^5^ Different therapeutic interventions have variable non-sustained results including topical steroids, intra-lesional triamcinolone, systemic antihistamines, corticosteroids or etidronate. Tacrolimus was found to be helpful in alleviating itching and hence improving quality of life.^6^ Excellent response is documented from systemic thalidomide perhaps due to its immunomodulatory action in a case report.^7^ Cryotherapy is certainly not a cure but beneficial in relieving pruritis.^8^ In the recent years, there are encouraging reports for the effectiveness of Dupilumab in reducing pruritis and improving skin findings. In future, it could be a new therapeutic option for patients of epidermolysis bullosa pruriginosa.^9^,^10^

## CONCLUSION

Unlike the typical cases of epidermolysis bullosa, the patients in this case report presented with intense pruritis in the absence of bullous lesions which was quite different as compare to most cases of EB, although family history was suggestive of genodermatosis. So, this case elaborates an uncommon presentation of EB. Also, it is need of the hour that in remote areas of developing countries where consanguineous marriages are exceedingly common, facilities of genetic councelling and pre natal testing for families affected by genetically transmitted diseases should be arranged on priority basis.
